# Geometry and Symmetry of Willis’ Circle and Middle Cerebral Artery Aneurysms Development

**DOI:** 10.3390/jcm13102808

**Published:** 2024-05-10

**Authors:** Carmelo Lucio Sturiale, Alba Scerrati, Luca Ricciardi, Oriela Rustemi, Anna Maria Auricchio, Nicolò Norri, Amedeo Piazza, Fabio Raneri, Alberto Benato, Alessio Albanese, Annunziato Mangiola, Donato Carlo Zotta, Giancarlo D’Andrea, Veronica Picotti, Antonino Raco, Lorenzo Volpin, Gianluca Trevisi

**Affiliations:** 1 Department of Neurosurgery, Fondazione Policlinico Universitario A. Gemelli IRCCS, Università Cattolica del Sacro Cuore, 00168 Rome, Italy; carmelo.sturiale@policlinicogemelli.it (C.L.S.); alessio.albanese@policlinicogemelli.it (A.A.); 2 Department of Translational Medicine, University of Ferrara, 44121 Ferrara, Italy; 3 Department of Neurosurgery, Sant’Anna University Hospital of Ferrara, 44121 Ferrara, Italy; 4 Neurosurgical Unit, NESMOS Department, Sapienza University of Rome, 00161 Rome, Italy; ricciardi.lu@gmail.com (L.R.); amedeo.piazza@uniroma1.it (A.P.);; 5 Department of Neurosurgery, San Bortolo Hospital, 36100 Vicenza, Italy; orielarustemi@libero.it (O.R.); fabran@gmail.com (F.R.);; 6 Department of Neurosurgery, University Medical Center Utrecht, 3584 CX Utrecht, The Netherlands; 7 Department of Neurosciences, Imaging and Clinical Sciences, G. D’Annunzio University, 66100 Chieti, Italy; 8 Neurosurgical Unit, Ospedale Spirito Santo, 65122 Pescara, Italy; 9 Neurosurgical Unit, Spaziani Hospital, 03100 Frosinone, Italy; giancarlo.dandrea@aslfrosinone.it (G.D.); picotti.1627238@studenti.uniroma1.it (V.P.)

**Keywords:** unruptured aneurysms, ruptured status, MCA geometry, hypoplasia

## Abstract

**Background**: A relationship between the geometry and symmetry of Willis’ circle and intracranial aneurysms was reported for anterior communicating and posterior communicating (PCom) aneurysms. A similar association with the middle cerebral artery (MCA) aneurysms instead appeared weaker. **Methods**: We reviewed 432 patients from six Italian centers with unilateral MCA aneurysms, analyzing the relationship between the caliber and symmetry of Willis’ circle and the presence of ruptured and unruptured presentation. CT-angiograms were evaluated to assess Willis’ circle geometrical characteristics and the MCA aneurysm side, dimension and rupture status. **Results**: The hypoplasia of the first segment of the anterior cerebral artery (A1) was in approximately one-quarter of patients and PCom hypoplasia was in almost 40%. About 9% had a fetal PCom ipsilaterally to the aneurysm. By comparing the aneurysmal and healthy sides, only the PCom hypoplasia appeared significantly higher in the affected side. Finally, the caliber of the internal carotid artery (ICA) and the first segment of MCA (M1) caliber were significantly greater in patients with unruptured aneurysms, and PCom hypoplasia appeared related to the incidence of an ipsilateral MCA aneurysm and its risk of rupture. **Conclusions**: Although according to these findings asymmetries of Willis’ circle are shown to be a risk factor for MCA aneurysm formation and rupture, the indifferent association with ipsilateral or contralateral hypoplasia remains a datum of difficult hemodynamic interpretation, thereby raising the concern that this association may be more casual than causal.

## 1. Introduction

**Figure 1:**
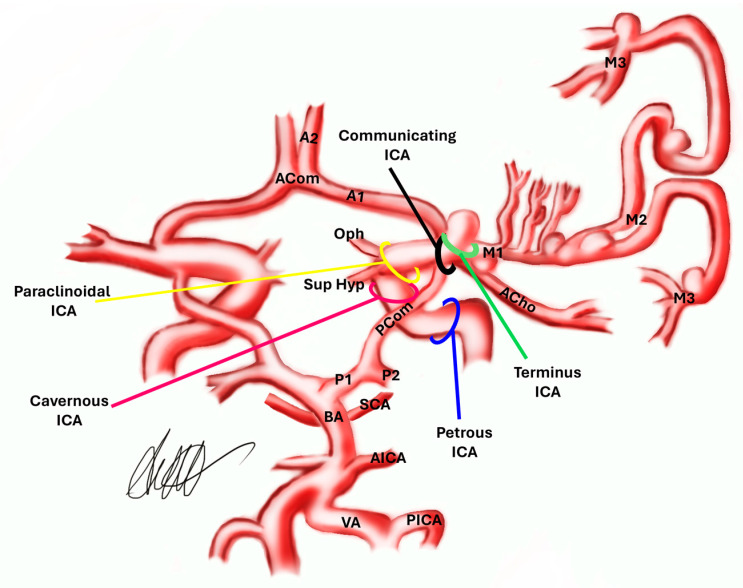
**Anatomy of the Willis Circle.** A1: anterior cerebral artery, tract 1; A2: anterior cerebral artery, tract 2; Acho: anterior choroidal artery; ACom: anterior communicating artery; AICA: anteroinferior cerebellar artery; BA: basilar artery; cavernous ICA: cavernous internal carotid artery; communicating ICA: communicating internal carotid artery; M1: middle cerebral artery, tract 1; M2: middle cerebral artery, tract 2; M3: middle cerebral artery, tract 3; Oph: ophthalmic artery; P1: posterior cerebral artery, tract 1; P2: posterior cerebral artery, tract 2; paraclinoidal ICA: paraclinoidal internal carotid artery; PCom: posterior communicating artery; petrous ICA: petrous internal carotid artery; PICA: posteroinferior cerebellar artery; SCA: superior cerebellar artery; Sup Hyp: superior hypophyseal artery; terminus ICA: terminus internal carotid artery.

After decades of debate, intracranial aneurysms (IAs) are by now mostly considered non-congenital vascular malformations, developing during life instead [1]. Accordingly, besides age, several other risk factors were identified that may favor their formation: among them, familial preponderance, polycystic kidney disease, atherosclerosis, smoking, and hypertension are known as the most important [1,2]. Some authors focused on the hemodynamic characteristics of Willis’ circle [3]. This ring-like structure formed via the internal carotid arteries (ICAs), anterior cerebral arteries (ACA), anterior communicating artery (ACom), posterior communicating arteries (PComs), and posterior cerebral arteries (PCAs), which are terminal branches of the basilar artery (BA), plays a vital role as a collateral circulation system that helps to maintain cerebral perfusion in the event of arterial occlusion (Figure 1). 

The circle of Willis shows frequent variations like hypoplasia, aplasia, or fenestrations of arteries, and can even retain fetal configurations of the posterior cerebral artery, potentially affecting blood flow [4]. Some studies demonstrated an association between the hypoplasia of some segments of the circle of Willis and the occurrence of IAs [5,6,7]. Three-dimensional computer simulation programs partially confirmed that parent vessel calibers, and in particular the hypoplasia, bifurcation angles, and asymmetry of the vascular tree, may enhance the shear stress and favor the development of the IAs [8,9,10,11,12,13]. A relationship between the geometry and symmetry of the Willis circle and aneurysm formation and rupture was frequently found for anterior communicating and posterior communicating artery aneurysms [14,15,16,17]. Instead, the data regarding a similar association with middle cerebral artery (MCA) aneurysm formation are weaker since these aneurysms occur outside of the anastomotic part of the Willis polygon and the role of the bifurcation morphology seems to be the most important predisposing factor [3,18]. In this study, we focused on a large multicenter series of patients with unilateral MCA aneurysms to analyze the relationship between the caliber and symmetry of the main segments of the Willis circle and the presence of aneurysms in this terminal branch.

## 2. Materials and Methods

### 2.1. Population

We retrospectively reviewed 432 consecutive patients admitted to six Italian tertiary referral cerebrovascular centers between 2015 and 2019 with a diagnosis of unilateral ruptured or unruptured MCA aneurysm. Patients with bilateral MCA aneurysms were excluded. CT-angiograms were retrieved from the institutional PACS, independently analyzed by the participant centers, and then collected in a single database. 

### 2.2. Measurements

**Figure 2:**
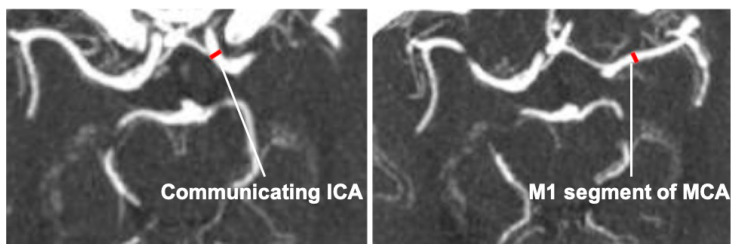
**Diameter measurements of major Willis circle segments.** Measurements were performed on TC-angiograms in the arterial phase using the maximum intensity projection (MIP) images.

For each patient, we assessed several available pieces of demographics, clinical, and angioarchitectural data. We postulated that the flow within the arterial tree of the MCA is directly proportional to the caliber, not only of the first segment of MCA (M1) but also of the main vessels of the Willis circle and particularly the internal carotid artery (ICA), the first segment of the anterior cerebral artery (A1) and the PCom. Moreover, as the Willis circle is an anastomotic system, both the ipsilateral and the contralateral Willis arteries may play a role in influencing the flow in one MCA, which in turn is a terminal branch of the same vascular system. In fact, if on one hand the flow of the ICA represents the main contribution to the ipsilateral Willis’ hemi-system, A1 tracts of a larger caliber (with patency of the ACom) or, on the contrary, the hypoplasia of the same segments, may influence the flow increasing or decreasing the interhemispheric blood flow shunt. A ratio of the width of the larger vessel’s segment to the corresponding contralateral smaller one was determined. Additionally, the presence of a fetal variant of the PCom artery may theoretically increase the ipsilateral MCA flow through the contribution of the posterior circulation. Therefore, we evaluated the relationship among the sides, dimensions, and rupture status of the MCA aneurysms and the symmetry and caliber of the main segments of the Willis circle. The diameter of the ICA was measured in the communicating tract process (also called the ICA communicating tract [19]) while the caliber of the MCA was calculated at the M1 segment (Figure 2). The PCom tract was defined as “fetal” when the proximal PCA (P1 segment) was hypoplastic or absent and PCom served as the main dominant source of blood supply to the PCA territory [20]. The vessel was considered hypoplastic when the width was less than 50% of the width of the contralateral corresponding larger vessel [21]. Measurements were performed on TC-angiograms in the arterial phase using the maximum intensity projection (MIP) images.

### 2.3. Statistical Analysis

We analyzed the main geometrical characteristics of the main circle of Willis vessels in the entire population; then, we compared them between the hemi-circulation harboring the aneurysm at the MCA bifurcation and the healthy side. We also sought a possible correlation between the size of the aneurysm and the diameter of the bilateral ICA and MCA and compared the aneurysm size between patients with and without A1 or PCom hypoplasia on either side as well as with the fetal origin of PCom on the aneurysm side. We compared the same angioarchitectural characteristics between ruptured and unruptured aneurysms. Lastly, we performed the above analyses in the subgroup of unruptured aneurysms to exclude the possible influence of radiological narrowing on the vessels’ caliber in the acute phase of ruptured aneurysms. Indeed, clinical vasospasm as delayed cerebral ischemia is a dynamic process that can be quantified definitively with transcranial Doppler and CT angiography, but this aspect occurs usually between 5 and 21 days after SAH. Therefore, the data retrieved for our measurements considered the CTA in the first hours after aneurysm ruptures. Student’s *t*-test, Pearson’s Chi-Squared test, and Pearson’s correlation coefficients were used as appropriate, with alpha set at 0.05. JASP 0.16.4 software was used.

## 3. Results

### 3.1. Demographic, Clinical, and Angioarchitectural Data of the Entire Population

We collected data from 432 patients harboring unilateral MCA aneurysms with a mean age of 58.5 ± 11.8 years. Almost three-quarters of patients were female (317; 73.4%) and more than one-third of the aneurysms (169; 39.1%) were ruptured upon clinical presentation. Most of them were located at the bifurcation (333; 77.1%) or at the origin of a precocious temporal branch (61; 14.1%); 33 aneurysms (7.6%), instead, originated from the M1 segment, 2 (0.5%) from the M2/M3 transition, and 3 (0.7%) from the M3 segment. The mean aneurysm size was 7.1 ± 4.2 mm, measured at the larger diameter. The main known risk factors for aneurysm formation and rupture such as smoking habits and high blood pressure were present in more than one-third (156; 36.1%) and about three-quarters (314; 72.7%) of cases, respectively (Table 1).

### 3.2. Association between MCA Aneurysm Occurrence and the Geometry and Symmetry of the Willis Circle

Concerning the geometry of the main segments of the Willis circle, we observed that the mean diameters of the ipsilateral and contralateral ICA were similar (3.7 ± 0.8 vs. 3.6 ± 0.7) as well as the mean caliber of the MCA (2.8 ± 0.7 vs. 2.7 ± 0.7), measured at the M1 segment, which appeared in general larger on the aneurysm side than the contralateral one, showing a statistical trend. PCom hypoplasia was significantly more frequent on the aneurysm side compared to the contralateral healthy side (26.8% vs. 12%; *p* < 0.001). Likewise, the rate of A1 segment hypoplasia appeared higher, although not significantly, on the aneurysm side (19.4% vs. 3.7%). Moreover, the comparison between the side with the MCA aneurysm and the healthy opposite side revealed no significant difference in the prevalence of A1 segment hypoplasia (Table 1 and Table 2).

### 3.3. Association between MCA Aneurysm Size and the Geometry and Symmetry of the Willis Circle

The size of the MCA aneurysms did not appear significantly related to the dimensional characteristics of ipsilateral and contralateral parent vessels, such as the communicating ICA and the M1 segment (Table 3). Similarly, no relationship was demonstrated between the aneurysm size and the hypoplasia of the ipsilateral or contralateral A1 segment as well as an ipsilateral fetal PCom artery or ipsilateral or contralateral PCom hypoplasia (Table 4). Also, in limiting the analysis to unruptured MCA aneurysms, no significant correlation was found between the aneurysm size and ipsi- or contralateral ICA and MCA diameters.

### 3.4. Association between Aneurysm Ruptured Status and the Geometry and Symmetry of the Willis Circle

Patients with ruptured and unruptured MCA aneurysms were comparable for mean age and main risk factors for rupture such as smoking and high blood pressure, whereas they differed in aneurysm size, as ruptured aneurysms were significantly larger than unruptured ones (7.7 vs. 6.7 mm, *p* = 0.02). These results are reported in Table 5.

Patients with ruptured aneurysms had on average a caliber of the ipsilateral ICA significantly smaller than the same segment in patients harboring unruptured aneurysms (*p* = 0.001). Similarly, the caliber of the ipsilateral MCA measured at the M1 segment was also significantly smaller than the same segment in patients harboring unruptured aneurysms (*p* = 0.001). On the other hand, the presence of ipsilateral or contralateral hypoplasia of the A1 segments did not appear to be associated with the ruptured status of the MCA aneurysms. Finally, as regards the caliber of the PCom segment, the presence of an ipsilateral fetal origin did not appear to be associated with the ruptured status of the MCA aneurysms; on the contrary, hypoplasia of the same segment appeared significantly more frequent in patients with ruptured MCA aneurysms both ipsilaterally and contralaterally (Table 6). 

## 4. Discussion

Our study explored the potential role of the main anatomic variations of the Willis circle as a risk factor for MCA aneurysm formation and rupture. The importance of this subject is given by the fact that the recognition of a relationship between the geometry and symmetry of the circle of Willis and the presence of aneurysms or their risk of rupture has a clinical impact in the planning of screening and follow-up protocols, especially in an era in which non-invasive neuroimaging diagnostics are extremely widespread and the detection of incidental aneurysms is continuously increasing [22]. 

An A1 segment hypoplasia was identified in approximately one-quarter of patients with MCA aneurysms; this prevalence represents a notably higher rate compared to the 2% reported in healthy populations [23]. Similarly, we found a notably high prevalence of unilateral PCom hypoplasia in nearly 40% of the patients with MCA aneurysms. This rate is considerably higher than the reported incidence of 20% in healthy individuals [24]. Finally, about 9% of patients had a fetal origin of the PCom segment ipsilaterally to the MCA aneurysm occurrence, which is in line with the expected rate from the literature [25] (Table 1). However, by comparing the side harboring the aneurysm with the healthy side, only the occurrence of a PCom hypoplasia appeared significantly higher in the affected side (Table 2). This finding seems to be of more of a casual than causal nature, as in a certain sense, it only proves the lack of a contribution to the flow from the posterior circulation to the hemi-system of the circle of Willis harboring the aneurysm. Similarly, neither the main geometrical characteristics, such as the caliber of these Willis circle segments, nor their symmetry seemed to influence the MCA aneurysm size (Table 3 and Table 4). Additionally, by comparing patients with ruptured and unruptured aneurysms, we observed that both the caliber of the ipsilateral and contralateral ICA and M1 segments of the unruptured aneurysm were significantly larger than those of the ruptured ones. In other words, the risk of rupture for the MCA aneurysms in this series seemed higher in patients with a Willis circle of smaller caliber. Nonetheless, we cannot rule out the possibility of some degree of radiological spasm as an acute vasomotor response responsible for the narrowing of vessel walls in the vasculature immediately adjacent to the aneurysm, after rupture. 

Lastly, our investigation identified a noteworthy association between PCom hypoplasia and the risk of MCA aneurysm rupture (Table 6). Interestingly, this association appeared to be even stronger for contralateral PCom hypoplasia compared to ipsilateral hypoplasia. While the underlying hemodynamic mechanisms influencing this laterality remain unclear, these findings warrant further exploration to elucidate whether there is a causal relationship with aneurysm rupture.

While our study did not identify a significant association between specific anatomical variation and MCA aneurysms, reporting these negative findings still offers some valuable clinical insights. This information can be used to refine risk stratification for individuals with identified anatomical variations on routine imaging, particularly those with a family history of intracranial aneurysms. By understanding which variations may not be significant risk factors, we can potentially reduce unnecessary long-term follow-up imaging and associated anxiety for these patients.

### 4.1. The Role of Willis Circle Anomalies in MCA Aneurysm Development

Variations in the Willis circle occur in approximately 60% of the general population [24]. The arteries that form this anastomotic circle may present variations in their geometry and symmetry and this may potentially generate changes in blood flow both in the anterior and posterior circulation. These hemodynamic alterations increase the stress on vessel walls, which in turn was demonstrated to be associated with the pathophysiology of IAs [3,26,27]. Tanaka et al. [26] demonstrated in 125 subjects, using two-dimensional cine phase contrast MRI, that flow rates in both the carotid and basilar arteries were significantly different with the presence of circle of Willis variations. Some authors showed through simulation studies an increase in hemodynamic stress associated with anatomic variants of Willis’ circle in those places where aneurysms are more frequent. In these studies, the flow distribution among cerebral arteries was assumed to be linked with the geometrical properties of the cerebral arteries [28,29]. Although the association of variations and aneurysms had been used in the past as an argument in favor of a congenital theory of aneurysmal development, today, the most accredited theory is the hemodynamic stress caused by these anatomical variations. While researchers have expressed great interest in the above-mentioned hemodynamic indices, they cannot easily be measured in vivo with the current techniques but can only be indirectly derived from simulations or the geometrical characteristics of Willis’ circle. This aspect makes it difficult to reach conclusions on all the derived hemodynamic indices since there is no universally accepted numerical model for cerebral simulation. Historically speaking, the first piece of evidence that there was a suspected association between anomalies in the circle of Willis and the incidence of aneurysms dates back a century ago [30,31,32,33]. Today, this relationship was successfully confirmed several times with ACom artery aneurysms [14,15,16,17,27,34,35], whereas relatively few reports exist regarding the association with those originating from MCA. In general, these aneurysms seem to develop more often in bifurcations with hypoplastic branches than in bifurcations with no hypoplastic branches. However, the development of aneurysms cannot be explained solely through an increased impingement of axial flow to an apex [36,37], and some other hemodynamic factors such as secondary factors flow as well as the local structure must be taken into consideration. Also, aneurysms develop more often in bifurcations with relatively sharp angles, where the bloodstream deviates significantly from the direction of flow of the parent artery, than in bifurcations with more obtuse angles, where the bloodstream deviates relatively little from the direction of flow of the parent artery [3,21,26]. This last geometrical characteristic would seem to be the most important predisposing feature compared with the circle of Willis’ symmetry for the risk of MCA aneurysm development, aneurysms that are often more similar to a bifurcation dysplasia than true saccular aneurysms [38,39,40].

### 4.2. The Role of Wills Circle Anomalies in MCA Aneurysm Rupture

If, on the one hand, the association between Willis circle variations and the presence of some IAs is well documented, there is still a lack of consistency in relation to the hemodynamic forces imposed on aneurysms and the risk of rupture. ACom and PCom artery aneurysms are the most common IAs, accounting for 23–40% and 15–20% of IAs, respectively, and are more likely to rupture than other types of IAs because of their anatomic and hemodynamic characteristics [41,42,43]. The asymmetry of the proximal segment of the ACA and fetal pattern of the PCom artery are more often found in patients with ruptured aneurysms in these locations [44,45,46]. The association between circle of Willis anomalies and aneurysm rupture in previous reports has been primarily descriptive [5,47,48,49] or consisted of much fewer cases. In the study by Lazzaro et al. [15], among ACoA and PCoA aneurysms, anomalies of Willis’ circle occur more frequently in patients with ruptured cerebral aneurysms than in those that have not ruptured. This finding suggests that the presence of Willis circle anomalies in association with ACom or PCom artery aneurysms may be also a risk factor for rupture. On the contrary, De Rooij et al. [47] reviewed the CT angiograms of 126 patients, looking at the incompleteness, asymmetry, or dominance of the circle of Willis configuration, and found no association with rupture on a per-site basis. However, their series included only 22 ACom and 20 PCom artery aneurysms, and the definition of A1 segment hypoplasia differed from most other studies. For different aneurysm locations, such as those located at the MCA bifurcation, the role of the hemodynamic changes due to the most common Willis’ circle appears less important as a pathophysiological feature. Maslehaty et al. [18] showed that the extra-aneurysmal flow dynamics were non-significant in MCA mirror aneurysm development, and the aneurysmal geometry also did not seem to play a role as a predictor for rupture. Accordingly, the increased flow through an ipsilateral fetal origin PCom artery may not constitute excessive hemodynamic stress on the MCA bifurcation, thus being implicated neither in the formation of aneurysms nor in their rupture. The literature, in fact, appears to lack similar reports for MCA aneurysms.

### 4.3. Pathophysiologic Considerations

The circle of Willis provides a vascular redundancy that optimizes blood flow to different brain areas [47]. This anastomotic structure enables an interhemispheric anterior circulation flow through the ACom artery and communication with the posterior circulation through the PCom arteries. The flow through these collateral pathways, especially in the case of asymmetry, determines a deviation from the geometric optimality principles that in certain bifurcations do not achieve minimal energy expenditure, determining vessel wall stress [6]. In fact, it has been clinically observed that most cerebral aneurysms are in areas with increased hemodynamic forces such as the apex of arterial bifurcations or on tortuous vessels, rather than along straight vessels [28]. This may explain the remarkable predictability of aneurysm location despite the wide variability in aneurysm size and morphology. Bor et al. [3] demonstrated the putative role of hemodynamics in IA formation by evaluating the neuroimaging of 26 patients who developed de novo aneurysms compared with 78 controls who did not develop an aneurysm during the follow-up. They found a significant association between de novo IA formation and hypoplastic branches in those bifurcations with sharp angles. This study first demonstrated the association between IA formation and cerebrovascular geometries that were measured prior to aneurysm development, making this hypothesis more consistent with a true risk factor. Moreover, Tanaka et al. demonstrated with phase-contrast MRA that ICA flow volume was increased in the presence of an ipsilateral fetal PCom artery and a contralateral absent A1 when compared with subjects with a normal circle of Willis configuration [26]. Therefore, on a theoretical basis, increased flow in combination with a predisposing vascular configuration that does not minimize energy expenditure at bifurcations may determine a turbulent flow and increase the wall shear stress, contributing to IA formation and rupture.

### 4.4. Limitations

We Recognize Several Limitations to Our Study.

First, we could not use a truly healthy population with no evidence of any intracranial aneurysms as a control. Instead, we compared Willis’ hemi-circle harboring the MCA aneurysm with the contralateral healthy side.

Secondly, the development of intracranial aneurysms likely results from multiple variables determining vasculopathy changes that were not fully considered in this study. While hemodynamic factors undoubtedly play a role, a solely hemodynamic analysis provides an incomplete picture of MCA aneurysm formation and rupture. Integrating biological, biochemical, and potentially even genomic data is crucial for a more comprehensive understanding of the underlying mechanisms.

Finally, when comparing ruptured and unruptured aneurysms, the measurement of the caliber of the major vessels could have been influenced by the occurrence of vessel narrowing (intended as an acute vessel’s wall reaction to the hemorrhage and not as delayed cerebral ischemia) in patients with subarachnoid hemorrhage (SAH).

## 5. Conclusions

This study did not establish a robust association between variations in geometry and symmetry of Willis’ circle and the incidence of MCA aneurysms. Nevertheless, a frequent association between the ruptured status of aneurysms and Willis’ circle branches of smaller caliber, along with hypoplasia of the PCom artery, regardless of laterality, was observed. These findings suggest a potential link between vessel morphology and aneurysm rupture.

However, the weak association of some of these variables and the lack of a distinction between ipsilateral or contralateral asymmetries make these data difficult for hemodynamic interpretation. Consequently, we caution against making neuroradiological follow-up recommendations solely based on Willis’ circle asymmetry. Moving forward, this represents a preliminary basis for a prospective, observational, and longitudinal study that could provide deeper insights into the role of Willis’ circle variations in the formation, size, and rupture risk of MCA aneurysms. By integrating hemodynamic, biological, and potentially genomic data, future research may offer a more comprehensive understanding of the multifaceted mechanisms underlying intracranial aneurysm pathogenesis and rupture.

## 6. Patients

All patients expressed their written consent to the treatment of their personal data for scientific purposes, contextually with the informed consent for surgery.

## Figures and Tables

**Table 1:** Demographic, clinical, and angioarchitectural data of the entire population.

	Patients (*n* = 432)
Age in years	58.5 ± 11.8
Female gender	317 (73.4%)
Male gender	115 (26.6%)
High blood pressure	314 (72.7%)
Smoking	156 (36.1%)
Ruptured presentation	169 (39.1%)
Mean aneurysm size in mm	7.1 ± 4.2
Mean ipsilateral ICA diameter in mm	3.7 ± 0.8
Mean contralateral ICA diameter in mm	3.6 ± 0.7
Mean ipsilateral MCA in mm	2.8 ± 0.7
Mean contralateral MCA in mm	2.7 ± 0.7
Ipsilateral A1 hypoplasia	84 (19.4%)
Contralateral A1 hypoplasia	16 (3.7%)
Ipsilateral fetal PCom origin	38 (8.8%)
Ipsilateral PCom hypoplasia	116 (26.8%)
Contralateral PCom hypoplasia	52 (12%)

**Table 2:** Comparison between the healthy side and the aneurysm side.

	Healthy Side	Aneurysm Side	*p*-Value
**Mean ICA diameter in mm**	3.6 ± 0.7	3.7 ± 0.8	0.3
**Mean MCA diameter in mm**	2.7 ± 0.7	2.8 ± 0.7	0.06
**A1 hypoplasia**	16 (3.7%)	84 (19.4%)	0.17
**PCom hypoplasia**	52 (12%)	116 (26.8%)	**<0.001**

**Table 3:** Correlation between aneurysm size and ipsi- or contralateral ICA and MCA diameters.

Correlation		Pearson’s r	Lower 95% CI	Upper 95% CI	*p*-Value
**Aneurysm Size**	**Age**	0.078	−0.019	0.173	0.11
	**Ipsilateral ICA diameter**	0.010	−0.090	0.109	0.85
	**Contralateral ICA diameter**	0.021	−0.079	0.120	0.67
	**Ipsilateral M1 diameter**	−0.030	−0.129	0.070	0.56
	**Contralateral M1 diameter**	−0.013	−0.113	0.086	0.79

**Table 4:** Aneurysm size: comparison between patients with or without Willis circle vessel variations.

Grouping Variable		Mean Aneurysm Size in mm	Standard Deviation	*p*-Value
**Ipsilateral A1 hypoplasia**	**No**	7	4.4	0.4
	**Yes**	7.5	3.9	
**Contralateral A1 hypoplasia**	**No**	7.1	4.3	0.9
	**Yes**	7.3	3.9	
**Ipsilateral fetal PCom origin**	**No**	7.1	4.4	0.8
	**Yes**	7.3	3.5	
**Ipsilateral PCom hypoplasia**	**No**	7	4.4	0.4
	**Yes**	7.4	3.9	
**Contralateral PCom hypoplasia**	**No**	7.1	4.3	0.6
	**Yes**	6.8	3.9	

**Table 5:** Comparison of clinical anamnestic variables between ruptured and unruptured MCA aneurysms.

	Unruptured *n* = 263 (60.9%)	Ruptured *n* = 169 (39.1%)	*p*-Value
**Age in years**	59.2 ± 10.5	57.2 ± 13.3	ns
**Size in mm**	6.7 ± 4	7.7 ± 4.4	**0.02**
**High blood pressure**	197 (74.9%)	117 (69.2%)	ns
**Smoking**	100 (38%)	56 (33.1%)	ns

**Table 6:** Comparison of vascular morphological variables between ruptured and unruptured MCA aneurysms.

	Unruptured *n* = 263 (60.9%)	Ruptured *n* = 169 (39.1%)	*p*-Value
**Ipsilateral ICA diameter in mm**	3.8 ± 0.8	3.5 ± 0.7	**<0.001**
**Contralateral ICA diameter in mm**	3.8 ± 0.7	3.5 ± 0.8	**<0.001**
**Ipsilateral MCA diameter in mm**	2.9 ± 0.7	2.6 ± 0.7	**<0.001**
**Contralateral MCA diameter in mm**	2.8 ± 0.7	2.6 ± 0.6	**0.003**
**Ipsilateral A1 hypoplasia**	51 (19.4%)	33 (19.5%)	ns
**Contralateral A1 hypoplasia**	10 (3.8%)	6 (3.5%)	ns
**Ipsilateral fetal PCom origin**	24 (9.1%)	14 (8.8%)	ns
**Ipsilateral PCom hypoplasia**	62 (23.5%)	54 (32%)	**0.04**
**Contralateral PCom hypoplasia**	17 (6.4%)	35 (20.7%)	**<0.001**

## Data Availability

The dataset that supports the findings of this study is available from the corresponding author, A.M.A., upon reasonable request.
